# Toxoplasma-induced behavior changes - is microbial dysbiosis the missing link?

**DOI:** 10.3389/fcimb.2024.1415079

**Published:** 2024-09-30

**Authors:** Emese Prandovszky, Emily G. Severance, Victor W. Splan, Hua Liu, Jianchun Xiao, Robert H. Yolken

**Affiliations:** Stanley Division of Developmental Neurovirology, Department of Pediatrics, School of Medicine, Johns Hopkins University, Baltimore, MD, United States

**Keywords:** toxoplasma, microbiome, behavior, neurotransmitters, immune activation

## Abstract

*Toxoplasma gondii* (*T. gondii)* is one of the most successful intracellular protozoa in that it can infect the majority of mammalian cell types during the acute phase of infection. Furthermore, it is able to establish a chronic infection for the host’s entire lifespan by developing an encysted parasite form, primarily in the muscles and brain of the host, to avoid the host immune system. The infection affects one third of the world population and poses an increased risk for people with a suppressed immune system. Despite the dormant characteristics of chronic *T. gondii* infection, there is much evidence suggesting that this infection leads to specific behavior changes in both humans and rodents. Although numerous hypotheses have been put forth, the exact mechanisms underlying these behavior changes have yet to be understood. In recent years, several studies revealed a strong connection between the gut microbiome and the different organ systems that are affected in *T. gondii* infection. While it is widely studied and accepted that acute *T. gondii* infection can lead to a dramatic disruption of the host’s normal, well-balanced microbial ecosystem (microbial dysbiosis), changes in the gut microbiome during the chronic stage of infection has not been well characterized. This review is intended to briefly inspect the different hypotheses that attempt to explain the behavior changes during *T. gondii* infection. Furthermore, this review proposes to consider the potential link between gut microbial dysbiosis, and behavior changes in *T. gondii* infection as a novel way to describe the underlying mechanism.

## 1 Introduction

Toxoplasmosis, caused by *T. gondii*, presents a significant global health concern, impacting approximately one-third of the world’s population (Pappas et al., 2009). While often asymptomatic, with an estimated 80 to 90% of acquired infections going unrecognized (Mittal and Ichhpujani, 2011), individuals with weakened immune systems, as well as the offspring of pregnant women, face an increased risk of severe toxoplasmosis (Wong and Remington, 1994; Wang et al., 2017). As a result, manifestations of toxoplasmosis range from mild symptoms to more severe outcomes, including congenital infection, miscarriage, chorioretinitis, and encephalitis (Dubey and Beattie, 1988; Halonen and Weiss, 2013), as well as associations with mental health conditions, such as anxiety (Markovitz et al., 2015), fear (Boillat, 2020), and schizophrenia (Torrey and Yolken, 2003; Yolken et al., 2009; Fuglewicz et al., 2017). Transmission of *T. gondii* occurs through congenital means, foodborne routes, zoonotic transmission, and, rarely, through organ transplant or blood transfusion from an infected donor (Hill and Dubey, 2002). The parasite’s ability to persist for extended periods, potentially throughout a lifetime, contributes to its widespread presence.

*T. gondii*, taxonomically classified as a coccidian, is a spore-forming, single-celled obligate intracellular organism. It undergoes both asexual and sexual reproduction, with asexual reproduction occurring in various host cells through cell division (Dubey and Beattie, 1988). Sexual reproduction is confined to the intestinal epithelium of Felidae family members, leading to the shedding of unsporulated oocysts in their feces (Dubey and Beattie, 1988). Genotyping data showed very limited sexual recombination between predominant *T. gondii* clonal lineages, namely Types I, II and III (Howe and Sibley, 1995), as well as non-clonal lineages, also called atypical strains (Shwab et al., 2014). Different *T. gondii* strains lead to different manifestations of the pathophysiology of the infection (Xiao and Yolken, 2015).

The tachyzoites, key players in acute infection, undergo rapid replication every 6 to 8 hours through endodyogeny. In the rodent host, *T. gondii* infection leads to severe inflammation of the small intestine, resulting in necrosis of mucosal villi and tissue destruction (Liesenfeld et al., 1996; Denkers, 2009). The parasite load correlates with the severity of this inflammation in the intestine (Dias et al., 2014). Chronic toxoplasmosis unfolds as a continuum (Cerutti et al., 2020) from acute toxoplasmosis into a persistent and latent state within immune competent individuals due to the host immune response (Dubey, 1998). During this transition from acute to chronic infectious stages, a slower replicating form (bradyzoites) of the *T. gondii* organism dominates. The bradyzoites are distinct in behavior and unlike tachyzoites, do not rupture host cells; instead, they form cysts, featuring a robust protective mechanism (Dubey et al., 1998). Cysts develop intracellularly and can persist for extended periods, potentially for the life of the host (Ferguson and Hutchison, 1987; Dubey et al., 1998). Brain, eyes, and muscles emerge as the primary sites for chronic, latent infection, although cysts have also been identified in various visceral organs, such as the kidney, liver, heart and lungs (Dubey et al., 1998), but parasite frequency is incredibly low. While maintaining ostensible dormancy in the host, there is a higher risk of reactivation in immunocompromised individuals (Daher et al., 2021). Despite their slow replication, bradyzoites exert a profound impact on neuronal structure and connectivity (Daher et al., 2021), as well as provoke brain-related immune responses (Xiao et al., 2016a; Li et al., 2019).

**Figure 1:**
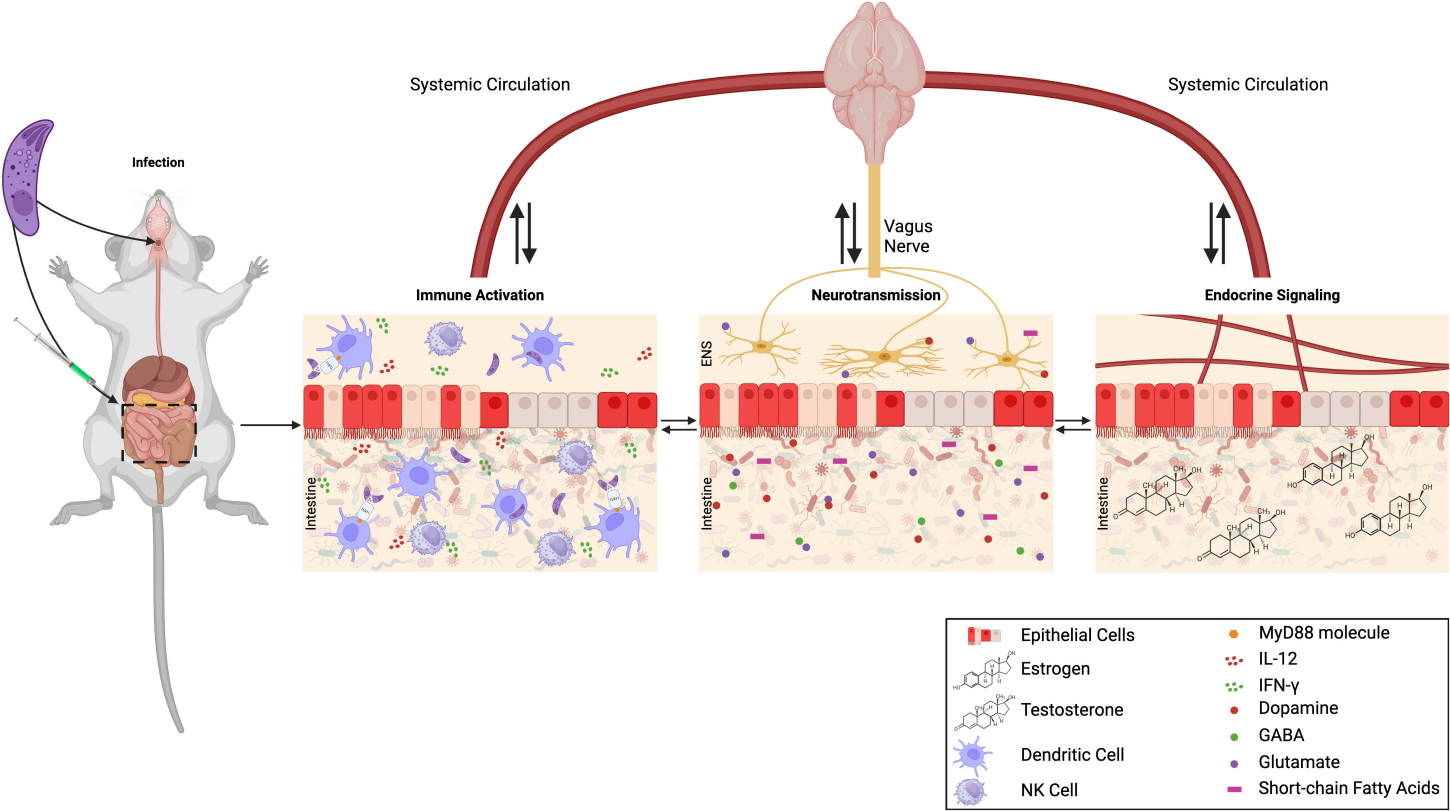
Working hypothesis - could the microbiome offer a precise piece of the puzzle to connect the different aspects of *T. gondii* infection that contribute to altered behavior? *T. gondii* infection (regardless of the infection route) affects the immune system, the nervous system, the endocrine system along with the gut microbiome. *T. gondii* infection leads to microbial dysbiosis in the intestine, depicted as red epithelial cells and microbes in the figure, as well as systemic immune activation, altered neurotransmission and endocrine signaling. Throughout the infection these organ systems interact with each other through the gut brain axis (GBA) which is bidirectional communication pathways. Created with BioRender.com.

Furthermore, *T. gondii* can directly affect host gene expression (Carruthers and Suzuki, 2007; Müller and Howard, 2016; Abo-Al-Ela, 2019) by influencing various host signal transduction pathways (Coppens and Joiner, 2001), neurotransmission (Stibbs, 1985; Adamec et al., 1998), host immune response (Hunter and Sibley, 2012; Yarovinsky, 2014; Sasai et al., 2018; Sasai and Yamamoto, 2019), and host behavior (da Silva and Langoni, 2009; Webster and McConkey, 2010; Pittman and Knoll, 2015; Vyas, 2015; Abdulai-Saiku et al., 2021; Tong et al., 2021). This intricate interplay defines the multifaceted nature of toxoplasmosis, revealing a dynamic, complex and enduring relationship between the parasite and its host. The most intriguing interplay between the parasite and host is the parasite manipulation of the host behavior. Despite numerous studies linking toxoplasmosis with behavior changes, no satisfactory stand-alone mechanism(s) responsible for these changes has been identified. In recent years the intestinal microflora has been receiving significant attention. Following a brief discussion of previous mechanistic hypotheses, we review the evidence that gut microbial dysbiosis, immune activation, neurotransmission, endocrine signaling, and behavior changes in *T. gondii* infection are linked and represent key features of a conceivable new underlying mechanism (**Figure 1**).

## 2 Previous hypotheses to explain chronic *T. gondii*-induced behavior changes

*T. gondii* has emerged as a significant focus of investigation due to its long-recognized capacity to manipulate the behavior of its hosts. Extensive research spanning decades has illuminated the intricate relationship between toxoplasmosis and behavioral alterations in a number of rodent (Berdoy et al., 2000; Gonzalez et al., 2007; Webster, 2007) and human studies (Flegr and Hrdy, 1994; Flegr et al., 1996). The behavioral manipulation hypothesis posits that *T. gondii* possess the ability to modify host behavior to their advantage (da Silva and Langoni, 2009; Webster and McConkey, 2010; Pittman and Knoll, 2015; Vyas, 2015; Abdulai-Saiku et al., 2021; Tong et al., 2021). The parasite’s manipulation of its host promotes the evolutionarily more advantageous sexual reproduction by enhancing the predation rates (Webster, 2007; Webster and McConkey, 2010). The most well-known behavior change induced by chronic *T. gondii* infection is the aversion to cat odors but no other predator odors (Berdoy et al., 2000). Despite rodents’ typical avoidance of areas with signs of cat presence, those infected with *T. gondii* exhibit a lack of fear, and in many instances, even show attraction to such areas (Berdoy et al., 2000). In mouse models, Torres et al. found that *T. gondii* infection induces anxiety-like behavior, alters spatial memory, and affects recognition of social novelty (Torres et al., 2018). Xiao et al. reported that infected mice exhibited reduced general locomotor activity, impaired object recognition memory, and lack of response to amphetamine trigger (Xiao et al., 2016b). All of the above cited works highlighted a different behavioral domain being affected in chronic *T. gondii* infection, suggesting that *T. gondii*-induced behavior changes may be variable and depends on both the host and parasite genetical background (Carruthers and Suzuki, 2007; Kannan et al., 2010; Xiao et al., 2012; Behnke et al., 2016).

The spectrum of behavioral changes associated with *T. gondii* infection extends beyond rodents to humans. Wong et al. reported that acute toxoplasmosis in immunocompetent adults may lead to moderately severe neurological symptoms (Wong et al., 2013). Congenital toxoplasmosis has been associated with diminished brain function and intellectual disability in humans (da Silva and Langoni, 2009). Chronic infection in men correlates with emotional instability and a disregard for social norms, while both infected men and women exhibit elevated anxiety levels (Flegr and Hrdy, 1994; Flegr et al., 1996; Flegr et al., 2000; Flegr et al., 2002; Flegr, 2007). Hinze-Selch et al. demonstrated in a study involving over 1000 subjects that toxoplasmosis can influence behavior and personality traits, particularly in psychiatric conditions (Hinze-Selch et al., 2010). The longstanding connection between toxoplasmosis and schizophrenia has been extensively documented (Torrey and Yolken, 2003; Mortensen et al., 2007; Henriquez et al., 2009; Yolken et al., 2009).

Despite numerous studies linking toxoplasmosis with behavior changes, no satisfactory explanation has been provided that can completely explain the mechanism(s) responsible for these changes. Below, we discuss the preexisting hypotheses in detail.

### 2.1 Immune activation hypothesis

The immune activation hypothesis emerged from the fact that *T. gondii* can induce an arsenal of immune responses upon infection (Yarovinsky, 2014; Sasai and Yamamoto, 2019). Briefly (Hunter and Sibley, 2012), at an early stage of the infection, the first responder host cells, in particular, dendritic cells (DCs), monocytes and macrophages, release proinflammatory cytokines. Toll-like receptors (TLRs) on these first responder cell types, have been described to have a central role in *T. gondii* antigen sensing. TLRs signal through myeloid differentiation primary-response gene 88 (MyD88), which is a central mediator of IL-12 secretion and the protective Th1 response to *T. gondii* in dendritic cells (Scanga et al., 2002). During the adaptive and innate immune response phases, IL-12 recruit interferon-γ (IFN-γ)-producing natural killer (NK) cells through the innate response, and CD4^+^ and CD8^+^ T cells through the adaptive response. The production of IFN-γ is responsible for activating cells to control parasite infection. For example, IFN-γ induces the production of nitric oxide (NO) and reactive oxygen species (ROS), both of which contribute to the control of intracellular parasite load in monocytes and macrophages. It also leads to a depletion of tryptophan and arginine, two amino acids, that are essential for *T. gondii* growth (Yarovinsky, 2014).

In acute infection, *T. gondii* induce monocytes and dendritic cell migration (Lambert et al., 2006) as well as promote the interruption of the blood-brain barrier, which allows the hijacked monocytes and dendritic cells to enter into the brain, carrying the parasite in a manner characterized as that of as a “Trojan horse” (Bierly et al., 2008; Lachenmaier et al., 2011). During acute infection parasites are observed infecting neurons, astrocytes, microglia, and infiltrating immune cells. Within the intricate landscape of the brain’s immune response, these resident cells play pivotal roles. Their contributions, marked by the production of chemokines, cytokines, and the expression of immune-regulatory cell surface molecules, collectively influence the dynamics of chronic toxoplasmosis (Daher et al., 2021). Astrocytes and microglia as well as peripheral monocytes can clear parasites through cell-autonomous immune pathways (Hunter and Sibley, 2012; Yarovinsky, 2014). Hence, as chronic infection progresses, cysts are primarily present within neurons. While immune cells in the brain contribute to *T. gondii* restriction, the role of cell-autonomous immunity in neurons is restricted by default to promote survival of neurons, which have an extremely limited regenerative ability. The immune response to *T. gondii* is sustained throughout chronic infection, resulting in elevated *T. gondii*–specific IgG and IFN-γ in the sera, both of which are essential to constrain the parasite growth and promote tachyzoite to bradyzoite conversion (Sturge and Yarovinsky, 2014; Zhao and Ewald, 2020).

Notably, the route of infection (intraperitoneal (IP) vs. oral) influences the sequential immune activation in rodents (French et al., 2022). The interaction of *T. gondii* antigens such as profilin with Toll-like receptor 11 (TLR11) on dendritic cells is important for host production of IL-12, especially in IP infection. For example, in mice that are infected IP with *T. gondii*, the parasite protein profilin directly binds and activates TLR11, contributing to IL-12 production and parasite restriction (Yarovinsky et al., 2005). However, profilin is an intracellular cytoskeletal protein that is crucial for movement and invasion of *T. gondii*. The fact that TLR11 receptors samples from endosomal compartments, implies that this pathway may be mostly activated by phagocytosed, dead, or dysfunctional parasites. In contrast, after oral infection in a TLR11-deficient mouse model, there were minimal defects in their Th1 response compared with mice deficient in MyD88 or other TLRs (Minns et al., 2006; Debierre-Grockiego et al., 2007; Denkers, 2009; Foureau et al., 2010; Zhao and Ewald, 2020). Of note, both IP and oral infection produce comparable behavior outcomes.

As described above, *T. gondii* is able to activate the immune system and induce a substantial immune response. Activating a robust immune response is critical to both host and parasite survival. Interestingly, an activated immune system during chronic toxoplasmosis may present as a low-grade, constant inflammatory comorbidity that can manifest in neurological symptoms and behavior changes in both rodents (Boillat, 2020) and humans (Yirmiya, 1997; Schmidt et al., 2010).

### 2.2 Neurotransmitter hypotheses

In this section, we provide an overview of the rationale supporting that altered neurotransmitter abundances and activities could explain *T. gondii*-induced host behavioral changes. In this context, we focus on dopamine (Prandovszky et al., 2011), glutamate (David et al., 2016; Kannan et al., 2016; Lang et al., 2018; Li et al., 2018), and GABA (Brooks et al., 2015; Kannan et al., 2016). Furthermore, there are reports that neurotransmitter release upon *T. gondii* infection happens in sex-dependent fashion in mice, thus adding another variable to consider (Gatkowska et al., 2013). We expand on the sex differences in the following section.

#### 2.2.1 Dopamine

The discovery of two *T. gondii* enzymes (TgAAH1 & TgAAH2) that closely resemble mammalian tyrosine hydroxylases, enzymes that catalyze the rate limiting step in dopamine synthesis, shook the scientific community (Gaskell et al., 2009). Elevated dopamine signaling has been linked to *T. gondii*-induced behavior changes, where haloperidol (dopamine antagonist) rescued the predator aversion behavioral phenotype in rodents (Webster et al., 2006). Higher dopamine concentrations were detected *in vitro* in *T. gondii* infected neuronal cells, as well as *in vivo* in *T. gondii* infected mice brains (Prandovszky et al., 2011). Moreover, the host enzyme, dopamine decarboxylase (DDC), that converts L-DOPA to dopamine was also colocalized with the parasite cyst in this model (Martin et al., 2015). However, reports on the impact of *T. gondii* infection on dopamine metabolism have been subject to disagreement. The field was hoping to get a definitive answer by utilizing a knockout *T. gondii* strain. However, knocking out one of the tyrosine hydroxylase genes (TgAAH2), which is highly active in the bradyzoite stage, did not eliminate the *T. gondii*-induced behavioral effect (Wang et al., 2015; McFarland et al., 2018). Notably, since no double knockdown mutant has yet been created, having at least one of these two enzymes expressed seems to be crucial for the parasite’s survival (McConkey et al., 2015; Wang et al., 2015) and cannot completely rule out the role of the TgAAH genes in *T. gondii*-induced dopamine escalation. The inability of other groups to replicate these findings with different mouse and parasite strains suggests that the genetic background of the host and parasite strain needs to be considered (Carruthers and Suzuki, 2007; Kannan et al., 2010; Xiao et al., 2012; Behnke et al., 2016). Remarkably, McConkey’s group found that in parallel with dopamine increase, there is a decrease in noradrenaline due to a *T. gondii*-induced downregulation in dopamine ß-hydroxylase (DBH), which is the key enzyme catalyzing the dopamine to noradrenalin conversion (Alsaady et al., 2019). Specifically, *T. gondii* infected cells release extracellular vesicles containing a DBH antisense lncRNA, which is complementary to the DBH gene’s promoter region and crosses the transcription start site (Tedford et al., 2023), preventing DBH transcription and subsequently contributing to dopamine increase. Interestingly, DBH regulation takes place in a sex specific fashion caused by an estrogen receptor binding response element at the 5’ flanking region of the DBH gene (Alsaady et al., 2019).

#### 2.2.2 Glutamate

Glutamate is the main excitatory neurotransmitter in the brain, and its receptor (N-methyl-D-aspartate receptor (NMDAR)) plays a crucial role in synaptic plasticity and cognition, including learning and memory progression. This receptor system also plays a pivotal role in glutamate excitotoxicity, when excessive glutamate causes neuronal dysfunction and degeneration (Lau and Tymianski, 2010). Several groups reported an excess of glutamate and compromised NMDAR signaling in *T. gondii* infection (David et al., 2016; Kannan et al., 2016; Lang et al., 2018; Li et al., 2018), suggesting an increased risk for excitotoxicity and contribution to cognitive impairment. Interestingly, NMDA receptors have been implicated in anxiety-like behavior (Adamec et al., 1998).

#### 2.2.3 GABA

On the other hand, gamma-aminobutyric acid (GABA) is the main inhibitory neurotransmitter in the human brain and derives from glutamate (Schousboe et al., 2014). Through GABAergic signaling, *T. gondii* can modify the motility of host cells, in particular dendritic cells and microglia cells, thus potentially increasing the systemic propagation of the parasite (Fuks et al., 2012; Kanatani et al., 2017; Bhandage et al., 2019). In addition, *T. gondii* infection appears to alter the inhibitory function of GABAergic signaling in mice through changes in the distribution of an enzyme (GAD67) that catalyzes GABA synthesis in the brain (Brooks et al., 2015; Kannan et al., 2016). GABA hypofunction could decrease GABAergic activity, and consequently, reduce neuronal inhibitory control and contribute to excitotoxicity.

Neurotransmitter alteration exerts a more localized effect on *T. gondii*-infected host brains. Regions in the brain crucial for fear and anxiety responses, such as the medial amygdala, basolateral amygdala, and ventral hippocampus, according to some groups, exhibit higher cyst density, highlighting their role in behavioral alterations (da Silva and Langoni, 2009; McConkey et al., 2013), while others have not found a correlation between cyst location and these particular areas (Berenreiterová et al., 2011; McConkey et al., 2013). Furthermore, behavioral changes persist even after parasite cysts are cleared from the brain, hinting that tropism alone may not wholly account for these alterations (Ingram et al., 2013), and the effect is more systemic than local (Abdulai-Saiku et al., 2021).

### 2.3 Endocrine hypothesis

In addition to rapid acting neurotransmitters, long-acting hormones also affect the brain (Tong et al., 2021). Tong et al. proposed that *T. gondii*’s presence in rat ejaculate, coupled with increased testosterone synthesis, contributes to behavioral changes (Tong et al., 2021). Arousal naturally deters innate fear, and a shift towards attraction potentially favors *T. gondii*’s transmission. Additionally, the involvement of dopamine and arginine vasopressin in inducing impulsivity and recklessness is suggested (Tong et al., 2021). Even though sex differences in behavior changes are well documented in *T. gondii* infection (Flegr et al., 2008; Xiao et al., 2012), testosterone increase cannot easily explain changes in female behavior. Female mice show reduced survival rates and lower cytokine levels in comparison to male mice during acute *T. gondii* infection (Roberts et al., 1995). With respect to female hormones, treatment with estradiol and estrogen increase the number of tissue cysts in brain of both male and female mice (Pung and Luster, 1986). While greater resistance to *T. gondii* infection was found in the gonadectomized mice than sham-operated controls of both sexes (Kittas and Henry, 1980). Overall, sex hormones may have a complex vital role in the manifestation of *T. gondii* infection including *T. gondii*-induced behavior changes.

While the above listed hypotheses address certain aspects of these behavioral changes, the exact mechanism is still yet to be elucidated. Interestingly, these previously examined hypotheses involving *T. gondii*-associated immune activation, neurotransmitter functions and endocrine dysregulation, are consistent with an increasingly scrutinized role of the microbiome and its resident microbiota in shaping these very same processes. Could this burgeoning field of microbiome research offer a potential novel link to consider between *T. gondii* infection and altered behavior?

## 3 *T. gondii*-induced microbial dysbiosis in acute and chronic infection

The metagenome, otherwise known as the microbiome, includes the entire genetic content of all microorganisms, including bacteria, fungi, and viruses, that live inside or on the surface of the host (Marchesi and Ravel, 2015). The potential role of the microbiome in health and disease has been receiving increasing attention in recent years (Pflughoeft and Versalovic, 2012; Jovel et al., 2018; Gomaa, 2020). Due to the extensive impact of the microbiome on host physiology, it is considered to be the newest organ system in the body (Baquero and Nombela, 2012). The microbiome exhibits species/strain-specific and sex-specific differences, both of which are frequently encountered in studies of *T. gondii* infection. Since the primary infection route of *T. gondii* is likely to be ingestion, among the different microbiome sites, the gut microbiome is the most involved. The mammalian intestine contains 5 major anatomical areas starting from the stomach and extending distally: the duodenum, jejunum, ileum, cecum, and colon. The first three areas make up the small intestine, while the last two make up the large intestine. These individual sections of the intestine differ in pH (Evans et al., 1988) and oxygen saturation (Zheng et al., 2015), as well as cell surface receptors (Hickey et al., 2023), thus providing a series of unique niches, for the resident microorganisms.

The gut microbiome interacts with the nervous system through the gut-brain axis (GBA). The GBA can be envisioned as a bidirectional multilane highway, where the lanes represent the different routes of communication, via nerves, hormones, and microbial metabolites (e.g., short-chain fatty acids) (Carabotti et al., 2015; Clapp et al., 2017; Dinan and Cryan, 2017; Silva et al., 2020a). Evidence of microbiota-GBA interactions comes from the association of gastrointestinal (GI) dysbiosis with central nervous system (CNS) disorders, including autism and anxiety-depressive behaviors (Carabotti et al., 2015; Clapp et al., 2017). Due to the robust interactions between the gut microbiome and the immune system, infection-induced immune activation can also lead to intestinal dysbiosis which can manifest as functional GI disorders (French et al., 2019; Tomal et al., 2023). While it is widely studied and accepted that acute *T. gondii* infection leads to substantial microbial dysbiosis in the gut, changes in the gut microbiome during the chronic stage of infection is poorly characterized and often debated by the scientific community (Taggart et al., 2022).

### 3.1 *T. gondii*-induced bacterial dysbiosis in the gut during the acute phase of infection

*T. gondii* invasion of the epithelium in the small intestine leads to intense inflammation, dysbiosis, and tissue necrosis followed by the spread of gut bacteria to peripheral organs and subsequent sepsis (Wang et al., 2019). Paneth cells, in the small intestine constitutively produce antimicrobial peptides and growth factors that help maintain the status quo between the host and the microbiota. Araujo et al. and Raetz et al. demonstrated that IFN-γ acts directly on murine Paneth cells via the m-TOR pathway, resulting in subsequent cell death (Raetz et al., 2013; Araujo et al., 2021). Since natural *T. gondii* infection mainly affects the small intestine and causes severe ileitis in susceptible mice, acute *T. gondii* infection has become a widely utilized model to study the type 1 (Th1) immune response in functional GI diseases like irritable bowel syndrome (IBS) and Crohn’s disease (Frank et al., 2007; Rolhion and Darfeuille-Michaud, 2007; Cohen and Denkers, 2015). Thus, the effect of acute *T. gondii* infection on the intestinal bacteriome has been well described (Heimesaat et al., 2006; Benson et al., 2009; McKnite et al., 2012; Raetz et al., 2013; Burger et al., 2018).

Altered taxonomical composition in the gut microbiome during acute *T. gondii* infection is marked by elevated abundance of Gram positive Bacteroidetes at the expense of decreased Firmicutes. Several studies have reported on the outgrowth of Gram negative Proteobacteria, such as *E. coli*, during acute *T. gondii* infection (Heimesaat et al., 2006; Craven et al., 2012; Molloy et al., 2013). Although Proteobacteria ssp. contribute to the development of ileitis (Heimesaat et al., 2006), their presence also magnifies the parasite-induced IFN-γ driven immune response in the gut. In so doing, the bacteria prevent a faster systemic dissemination of *T. gondii* which protects the host in the long run (Benson et al., 2009). In germ-free mice, where commensal bacteria are not present, *T. gondii* infection resulted in systematic inflammation that was not constrained to the intestine (Nascimento et al., 2017).

The effect of acute *T. gondii* infection on the gut microbiome can have varying outcomes. The results highly depend on the following: 1) the genetic background of the host and the parasite, 2) the host age and gender, 3) the route of infection, and 4) the anatomical location of where the samples were collected from within the intestine (**Table 1**). For example, Lv et al. reported significantly higher bacterial diversity (species richness) in the fecal pellet of Wistar rats infected with a PSY strain of *T. gondii* (Lv et al., 2022), whereas utilization of the PRU strain instead of PYS showed a lower trend, but no significant differences in bacterial diversity compared to uninfected animals in concordance with others (Prandovszky et al., 2018; Shao et al., 2020; Lv et al., 2022; Meng et al., 2023). The plausible explanation of this difference might be the use of an atypical strain (PYS) in Lv’s study that produced a more robust infection, compared to the other studies where more common *T. gondii* strains were utilized.

**Table 1:** Acute dysbiosis.

T. gondii Strain	Rodent Strain* and Sex	Age at infection	Infection Route	Sample Origin	Acute Dysbiosis	Citation
GT1 (Type I)	CD1 ♂	6–8 weeks	IP	jejunal-ileal contents	↑ Bacteroidetes Beta diversity – significant community dissimilarities	(Prandovszky et al., 2018)
ME49 (Type II)	C57BL/6 ♂/♀?	2-4 months	Oral	ileal contents	↑ Enterobacteriaceae/*E.coli* ↑ *Bacteroides/Prevotella* spp. ↓ Lactobacilli ↓ Clostridia ↓ Alpha diversity	(Heimesaat et al., 2006)
ME49 (Type II)	C57BL/6 ♀	12 weeks	Oral	ileal contents	↑ γ-Proteobacteria (*E.coli*) ↓ Firmicutes ↓ Alpha diversity	(Craven et al., 2012)
ME49 (Type II)	C57BL/6J ♀ BALB/cJ ♀	9-10 weeks	Oral	ileal contents	Only in C57BL/6J mice: ↑ γ-Proteobacteria/Enterobacteriaceae ↓ Fusobacteria ↓ Alpha diversity	(Wang et al., 2019)
ME49/C1 (Type II)	C57BL/6 ♀/♂?	8-12 weeks	Oral	colon content & small intestinal mucosa associated bacteria	↑ γ-Proteobacteria/Enterobacteriaceae ↓ Firmicutes/Clostridia	(Molloy et al., 2013)
ME49 (Type II)	C57BL/6 ♀	3 weeks	Oral	ileum	↑ Enterobacteriaceae (*E.coli*) ↑ *Bacteroides/Prevotella* spp. ↑ Enterococci ↓ Lactobacilli	(Haag et al., 2012)
ME49 (Type II)	C57BL/6 ♀	3 months	Oral	small intestine	↑ Enterobacteria, ↑ Enterococci, ↑ *Bacteroides/Prevotella* spp.,	(Bereswill et al., 2014)
PRU (Type II)	C57BL/6 ♀	8-10 weeks	Oral	cecum	↑ Acutalibacteraceae, ↑ Bacteroidaceae, ↑ CAG-465 (Clostridia), ↑ Gastranaerophilaceae ↓ Burkholderiaceae ↓ Clostridiaceae ↓ Alpha diversity Beta diversity – significant community dissimilarities	(Meng et al., 2023)

*Mice, unless otherwise indicated. ♀ indicates female, ♂ indicates male; ↑ indicates an increase in relative abundance, ↓ indicates a decrease in relative abundance.

### 3.2 *T. gondii*-induced bacterial dysbiosis in the gut during the chronic phase of infection

While it is widely studied and accepted that acute *T. gondii* infection leads to substantial microbial dysbiosis in the gut, changes in the gut microbiome during the chronic stage of infection are not well characterized. Some groups reported that as inflammation resolved in the ileum, and the infection progressed from the acute to the chronic stage, Firmicutes became the most abundant phylum, accompanied by a decrease in the abundance of Proteobacteria, the Gram negative taxa that led to substantial dysbiosis and intestinal pathology (Shao et al., 2020; French et al., 2022; Yan et al., 2022). Even if changes between IP and per oral infection in bacterial composition were similar, meaning the same bacterial phyla were affected but in different ratios, the route of infection can leave a distinctive imprint on the microbial community (French et al., 2022). As inflammation resolves in the ileum, the infection progresses from the acute to the chronic stage, giving the impression that the bacterial microflora in the intestine is restored. Yet, Hatter et al. found that changes in the commensal populations, notably an outgrowth of Clostridia spp., were sustained in *T. gondii* chronic infection (Hatter et al., 2018). Several studies also reported an enrichment in the taxa Verrucomicrobia (Akkermansiaceae) in animals with chronic *T. gondii* infection (McConkey et al., 2015; Prandovszky et al., 2018; Shao et al., 2020; Yan et al., 2022; Meng et al., 2023). This finding was universally present regardless of the sex and genetic background of the host, the route of infection, or the type of parasite strain; however, noticeable individual differences between animals were also observed. These findings suggest that changes in the composition of the gut microflora are not limited to the acute phase of *T. gondii* infection when the parasite induced pathology mainly localizes to the intestinal tract.

Interestingly, in numerous studies, bacterial diversity in the gut during chronic *T. gondii* infection was similar to acute infection, where infected animals showed lower bacterial diversity compared to uninfected controls (Shao et al., 2020; French et al., 2022; Lv et al., 2022; Meng et al., 2023). On the other hand, Prandovszky et al. showed significantly higher bacterial diversity in infected animals (Prandovszky et al., 2018). It’s of note that Prandovszky et al. used males in the experiment while all of the other groups used females.

The aforementioned studies collectively indicate that chronic *T. gondii* infection does indeed induce long-term microbiome changes in the gut, which are different from the acute stage of *T. gondii* infection. Although, in many instances, there is no clear concordance in changes affecting the bacterial microbiome, the variances likely depend on 1) the genetic background and sex of the host, 2) the host age, 3) the route of infection, and 4) the anatomical location of where the samples were collected from within the intestine (**Table 2**). Furthermore, understanding the microorganisms role are ultimately more important than simply running a taxonomical inventory. Gaining a truly meaningful insight to the function of the gut microbiome calls for species/strain level metagenomics sequencing of the intestinal content accompanied by metabolomics analysis of the gut and blood of the host.

**Table 2:** Chronic Dysbiosis.

*T. gondii* Strain	Rodent Strain* and Sex	Age at infection	Infection Route	Sample Origin	Chronic Dysbiosis	Citation
GT1 (Type I)	CD1 ♂	6–8 weeks	IP	small intestine	Lactobacillales (Cohort2) Bacteroidales (Cohort 2) ↑ Verrucomicrobia/Akkermansia (Cohort 1) ↑ Bacteroidetes (Cohort 1) ↑ Alpha diversity Beta diversity – significant community dissimilarities	(Prandovszky et al., 2018)
ME49 (Type II)	BALB/c ♀	6 weeks	Oral	cecum	↑ *Lactobacillus* ↑ R*ikenella* ↑ *Odoribacter* ↓ *S24-7*, ↓ Clostridiales, ↓ Desulfovibrio, ↓ Lachnospiraceae ↑ Alpha diversity Beta diversity – significant community dissimilarities	(Shao et al., 2020)
MeE9-GFP/luc (Type II)	C57BL/6J ♂	8-10 weeks	Oral	fecal pellet	↑*Clostridia* spp. Beta diversity – significant community dissimilarities	(Hatter et al., 2018)
ME49 (Type II)	C57BL/6J ♀	8-10 weeks	Oral (p.o.) vs. IP	ileum	↓ Bacteroides/Prevotellaceae ↑ Lactobacilliaceae (p.o.) ↓ Clostridiaceae ↓ Ruminococcaceae ↑ Erysipelotrichaceae (i.p.) ↑ Lachnospiraceae (i.p.) ↓ Alpha diversity (in p.o.) Beta diversity – significant community dissimilarities (in p.o. & i.p.)	(French et al., 2022)
PYS (Atypical) PRU (Type II)	Fischer/344 rats, ♀	4-6 weeks	IP	fecal pellet	↑ Verrucomicrobiaceae/Akkermansia ↓ Muribaculaceae ↓ Lachnospiraceae ↓Alpha Diversity Beta diversity – significant community dissimilarities	(Lv et al., 2022)
PRU (Type II)	BALB/c ♀	6 weeks	Oral	fecal pellet	↑Firmicutes ↓ Fusobacteria ↓ Bacteroidetes ↑ Alpha diversity Beta diversity – significant community dissimilarities	(Yan et al., 2022)
PRU (Type II)	Wistar Hannover rats ♂&♀	8 weeks	IP	cecum	No effect on alpha or beta diversity No differentially abundant taxon.	(Taggart et al., 2022)
PRU (Type II)	C57BL/6 ♀	8-10 weeks	Oral	cecum	↑ Atopobiaceae ↑ Burkholderiaceae ↑ Enterobacteriaceae ↑ Erysipelotrichaceae ↑ Muribaculaceae ↑ Pasteurellceae ↓ Desulfovibrionaceae ↓ Lachnospiraceae ↓Marinifilaceae ↓ Oscillospiraceae ↓ Anaeroplasmataceae ↑ Alpha diversity Beta diversity – significant community dissimilarities	(Meng et al., 2023)

*Mice unless otherwise indicated. ♀ indicates female, ♂ indicates male; ↑ indicates an increase in relative abundance, ↓ indicates a decrease in relative abundance.

Due to the pathological impact of *T. gondii* infection on the gut, microbiome studies are mainly profiling the ileum, and very few studies are assessing the large intestine (**Table 2**), where microbial metabolites, such as short chain fatty acids (Silva et al., 2020b), and dopamine as well as other neurotransmitters (Eisenhofer et al., 1997; Asano et al., 2012) are being produced. These microbial metabolites can have a critical influence on the brain (Needham et al., 2020). More research is needed to systematically examine and sample each section of the gut in a longitudinal fashion to reveal common trends and differences. Furthermore, this sampling should be implemented across multiple hosts with different genetic backgrounds and sexes as well as utilizing different parasite strains so that the effects of chronic *T. gondii* infection on the gut microbiome can be fully characterized. Studies utilizing different infection routes, will help in the understanding of the interplay between gut commensals and the immune activation in the behavioral outcomes.

The above-mentioned studies collectively underscore the complexity of microbiota-GBA interactions and shed light on the dynamic interplay between infectious agents, gut microbiome composition, and neurological outcomes in chronic infection. Further exploration of these relationships holds promise for advancing our understanding of the intricate mechanisms governing gut-brain communication in underlying *T. gondii*-induced behavioral traits (O'Mahony et al., 2009; Cryan and Dinan, 2012).

## 4 Connecting the dots between the different mechanistic approaches of *T. gondii* induced behavior and bacterial dysbiosis

The behavioral changes induced by *T. gondii* have been a focal point of research, with rodents and humans showing altered behavior that put the host at risk and provide survival advantages for the parasite. While the direct infection of the CNS and the parasite’s tropism to immune-privileged organs are notable considerations, the underlying mechanisms driving these changes remain unclear. Neuroinflammation, changes in neurotransmission, and endocrine signaling stand as pieces of the puzzle, awaiting precise connections, connections that the gut microbiome and GBA may be able to clarify. The GBA serves as a vital conduit for communication between the microbiome, gut, and brain, orchestrating an array of interactions through immune, neural and endocrine routes.

### 4.1 Bacterial dysbiosis impact on the immune activation

There is a well-established relationship between the immune system and the gut microbiota (Hooper et al., 2012). We described earlier the immune response to T. gondii in acute and chronic infection, focusing on MyD88 and TLR11, the main TLR that expressed on rodent dendritic cells, but not present in human macrophages. We pointed out, that in TLR11-deficient mice, the Th1 was not affected as significantly compared with mice deficient in MyD88 or other TLRs. What we haven’t mentioned, treating TLR11-deficient mice with antibiotics showed the same phenotype as MyD88-deficient mice (Flegr et al., 2000; Flegr et al., 2002) suggesting that commensals have a pivotal role in inducing Th1 immune response.

The immune response to toxoplasmosis involves a complex interplay of cytokines, chemokines, and lymphoid cells, with IFN-γ playing a central role in activating the host’s defense mechanisms, which involves a parasite-induced dysbiosis in the gut bacterial microflora, in particular Proteobacteria outgrowth, to combat *T. gondii* infection at the early stage (Benson et al., 2009). In germ-free mice, where commensal bacteria is not present, *T. gondii* infection resulted in systematic inflammation that was not constrained to the intestine (Nascimento et al., 2017).

Due to an intricate and continuous communication between the gut microbiota and the immune system, the outcome of this conversation does not leave the neurosystem unaffected and chronic changes in this communication can manifest in behavior traits.

### 4.2 Bacterial dysbiosis impact on neurotransmission

GBA profoundly influences central processes such as neurotransmission and behavior (Sherwin et al., 2016). Gut bacteria contribute to this dialogue by releasing an array of neuroactive compounds such as dopamine, glutamate, GABA (Lyte, 2011). These neurotransmitters seem to play an important role in microbial ecology (Lyte, 2013; Wall et al., 2014; Hulme et al., 2022), adding complexity when it comes to understanding the exact mechanisms by which intestinal microbes communicate with the CNS during *T. gondii* infection. Nevertheless, the evidence does strongly support that gut symbionts are key to CNS function, and therefore, alterations in their composition, diversity, and richness may play a role in the pathophysiology of the CNS during *T. gondii* infection.

#### 4.2.1 Dopamine

Alterations in dopaminergic transmission have been related to severe CNS disorders, such as anxiety (Zweifel et al., 2011; Zarrindast and Khakpai, 2015). Alteration of the gut microbiota impact dopamine signaling in the hippocampus and the amygdala (González-Arancibia et al., 2019). The dorsal hippocampus is a center of learning, memory, and spatial navigation in the brain, while the ventral hippocampus is associated with the emotional and motivational consequences of stress, including depression and anxiety (Bagot et al., 2015). The hippocampus communicates with subcortical structures, like the amygdala and the striatum. The amygdala is also involved in emotional behavior, participates in fear modulation, fear-associated memory, and attention (Phelps, 2004; Roozendaal et al., 2009), behavior traits that are associated with *T. gondii* infection.

In the absence of intestinal microbes, Heijtz et al. observed higher dopamine metabolism (increased dopamine turnover) in the striatum of adult germ free mice compared to specific pathogen-free (SPF) controls (Heijtz et al., 2011). Furthermore, they found that germ-free mice had higher expression of D_1_ mRNA expression in the hippocampus, while lower levels of expression of this receptor were found in the striatum when compared to SPF controls (Heijtz et al., 2011). In addition, germ-free mice showed fewer anxiety-like behaviors in comparison to their respective controls (Heijtz et al., 2011), while Nishino et al. observed the reversal of these outcomes perhaps due to the use of different mice strains in each study (Nishino et al., 2013).

Among several bacteria, *E. coli* has been reported to produce dopamine in the gut (Cryan and Dinan, 2012). It is known that acute *T. gondii* infection leads to Proteobacteria outgrowth, which in oral infection is likely to be *E. coli.* Excess of *E. coli* could contribute to the production of excess of dopamine in the gut. Excess dopamine can go through autooxidation that leads to the generation of reactive oxygen or nitrogen species (Meiser et al., 2013) which in turn might contribute to the necrotic pathology of the small intestine during acute toxoplasmosis (Liesenfeld et al., 1996; Denkers, 2009; French et al., 2022). Excess of dopamine can also signal the CNS through the gut-brain axis (González-Arancibia et al., 2019). On the other hand, Hatter et al. reported *Clostridia* outgrowth during chronic *T. gondii* infection (Hatter et al., 2018). Notably, metabolites produced by pathogenic *Clostridia* spp. can inhibit the conversion of dopamine to norepinephrine (Shaw, 2004; Shaw, 2017) resulting in elevated dopamine levels (Hamamah et al., 2022).

Other evidence for a complementary role of gut microbes in T. gondii infection comes from studies of probiotics such as *Lactobacillus rhamnosus* JB-1, *Bifidobacterium longum* NCC3001 in mice, and *Lactobacillu*s *helveticus* R0052 and *B. longum* R0175 in rats. These probiotic cocktails were found to reduce anxiety-like behavior in both models (Bercik et al., 2011; Bravo et al., 2011; Messaoudi et al., 2011). Moreover, administration of Lactobacillus plantarum PS128 to germ-free mice decreases anxiety-like behaviors, and these changes were accompanied by an increase in dopamine and homovanilicacid, as well as an increase in 5-HT in the striatum (Liu et al., 2016). Many groups have also detected *Akkermansia* enrichment in the gut of chronically infected mice (McConkey et al., 2015; Prandovszky et al., 2018; Shao et al., 2020; Yan et al., 2022; Meng et al., 2023). *Akkermansia* has been considered a next generation probiotic (Zhai et al., 2019), one that may have significant impact on the brain (Xu et al., 2023). If chronic *T. gondii* infection leads to an outgrowth of a bacteria with probiotic potential in the gut that would probably affect the brain.

#### 4.2.2 Glutamate

In the GI tract, dietary glutamate not only serves as a major source for glutamate, but it is also the most abundant (8%–10%) among dietary amino acids (Tomé, 2018). Several *Lactobacillus* strains have the ability to produce glutamate, with many of these *Lactobacillus* strains representing environmental bacteria or strains used in food fermentation (Sanchez et al., 2018). Interestingly, chronic *T. gondii* infection has also been linked to Lactobacilli overgrowth in mice (Prandovszky et al., 2018). About 75%–96% of enteral glutamate is removed during portal circulation in both humans and rodents for the production of energy (Haÿs et al., 2007). The brain is not exposed to an excess of glutamate due to low concentrations of glutamate reaching systemic circulation (Tomé, 2018). While dietary glutamate does not cross the blood brain barrier under normal conditions (Brosnan et al., 2014), an altered gut microbiota can cause changes in barrier permeability, as we saw with *T. gondii* infection, which can compromise the barrier and lead to the transfer of luminal glutamate into the CNS (Braniste et al., 2014; Kelly et al., 2015; Mazzoli and Pessione, 2016). The concentration of glutamate in neuronal cytoplasm is about 5 mM. However, the concentration of glutamate in astrocytes is lower (around 2–3 mM), and this is due to the function of an intact blood brain barrier. Excitatory amino acid transporters (EAAT) actively remove glutamate from the synaptic cleft and transport glutamate into the cytosol. Among these EAAT are GLAST and GLT-1, which are both expressed readily by astrocytes and are also both expressed by neurons and endothelial cells in the brain, although to a lesser extent. The homeostatic control of extracellular glutamate prevents its accumulation, as excess extracellular glutamate results in excitotoxicity (Pál, 2018), including excessive postsynaptic excitation (Brassai et al., 2015; Miladinovic et al., 2015), which has been linked to chronic inflammation (Kaszaki et al., 2012; Miladinovic et al., 2015). In addition, several groups have reported that there is an excess of glutamate, attributable to an impaired GLT-1 transport, and compromised glutamate receptor signaling during *T. gondii* infection (David et al., 2016; Kannan et al., 2016; Lang et al., 2018; Li et al., 2018). This suggests that there is an increased risk for glutamate-mediated excitotoxicity during *T. gondii* infection, which can potentially contribute to cognitive impairment.

Permeability to glutamate has been shown to increase in pathological conditions, such as in irritable bowel syndrome (IBS) and in inflammatory bowel disease (IBD). This increase in glutamate permeability has been shown to result in altered neuronal responses that are not only local in the enteric nervous system, but also remotely in the CNS by utilizing the microbiota-gut-brain axis. Elevated psychiatric co-morbidity in the development of IBS and IBD may be explained by impaired glutamate metabolism across the microbiota-gut brain axis (Holtmann et al., 2016; Mikocka-Walus et al., 2016). Notably both of these functional GI disorders have been modeled by *T. gondii* infection.

#### 4.2.3 GABA

GABA is the main inhibitory neurotransmitter in the CNS, and its imbalance has been associated with a number of disorders, including anxiety (Nuss, 2015).

Some gut commensal bacteria produce GABA including *Bacteroides*, *Bifidobacterium*, *Lactobacillus* species (Strandwitz et al., 2019; Barrett et al., 2012). Most bacteria, including *Lactobacillus* and *Bifidobacterium* genera producing GABA use the glutamate decarboxylase (GAD) pathways, while others such as *E. coli* can utilize both putrescine glutamate decarboxylase pathways (Diez-Gutiérrez et al., 2020). *T. gondii* infection appears to alter the inhibitory function of GABAergic signaling in mice through changes in the distribution of an enzyme (GAD67) that catalyzes GABA synthesis in the brain (Brooks et al., 2015; Kannan et al., 2016). GABA hypofunction could decrease GABAergic activity, and consequently, reduce neuronal inhibitory control and contribute to excitotoxicity. Yet, GABA-producing *Bifidobacterium adolescentis* strain reduced serum glutamate levels in mice (Royo et al., 2023).

Some experimental evidence indicates that the gut microbiome affects the level of GABA and subsequently influences mental health. For instance, Bravo et al. reported that *L. rhamnosus* elevated the abundance of GABA_B1b_ mRNA while decreasing the level of GABA_Aα2_ mRNA in the cortex of mice, leading to the inhibition of anxiety (Bravo et al., 2011; Terunuma, 2018).

The tenth cranial nerve (n. vagus), with its afferent fibers diligently detects metabolites, including neurotransmitters, produced by the microbiome in the gut, and conveys this critical information towards the CNS (Bonaz et al., 2018). Exploring the role of the vagus nerve during *T. gondii* infection holds potential opportunity for understanding how *T. gondii* utilize this neural materialization of the gut brain axis to influence behavior. Experimental vagotomy could offer crucial insights into the mechanisms linking toxoplasmosis to behavior changes. This exploration may not only illuminate the pathophysiology of toxoplasmosis but also underscore the role of the microbiome in mediating these complex interactions.

### 4.3 Bacterial dysbiosis impact on the hormone system

The neuroendocrine system and microbiome interact to influence social behaviors (Sylvia and Demas, 2018). Males and females exhibit distinct patterns in energy and nutritional requirements across the lifespan. Differences in sex hormones can contribute to differences in microbial diversity and gut microbial composition (Neuman et al., 2015). Collden et al. found higher levels of glucuronidated testosterone and dihydrotestosterone but lower levels of free dihydrotestosterone in the large intestine of germ-free mice compared with conventional mice that have a normal gut microflora, which suggests that the gut microbiome plays a crucial role in testosterone metabolism in mice (Colldén et al., 2019). Shin et al. reported that sex steroid hormone levels were correlated with diversity and gut microbial composition in humans, suggesting a robust communication between the two organ systems (Shin et al., 2019). Because it is a bidirectional communication, not only do sex hormones influence the intestinal microbiome, but the gut microbiota itself also influences hormone levels. Consistent with this activity researchers have observed a decline in estrogen levels during antibiotics treatment (Adlercreutz et al., 1984). Recently it has been shown that sex differences in the microbiome present an increased risk for developing autoimmune disorders in female mice, whereby fecal transfer of male intestinal microbiota to recipient females delayed onset and lessened severity of disease (Markle et al., 2013; Yurkovetskiy et al., 2013).

These findings suggest that epigenetic factors, such as infection, may induce sex-specific alterations in the composition of the gut microbiome, that contribute to sex differences in disease risk, since these epigenetic factors are known to lead to sex-specific alterations in immune function, metabolism, stress responsiveness and behaviors (O'Mahony et al., 2009; Cryan and Dinan, 2012).

As we explore the mechanisms by which acute and chronic *T. gondii* infection might lead to dysfunctional neural and glial circuitries, a role for the gut microbiome in the context of the gut-brain axis becomes apparent. The collective, balanced gut microbiome provides a variety of benefits and functions integral to human health and physiology (El Aidy et al., 2014; Dinan and Cryan, 2017). When awry, however, a pathological cycle mediated by ongoing, low-level inflammation fuels imbalances and produces a chronic state of translocated microbial communities that can impact the CNS (Martel et al., 2022; Jensen et al., 2023). Up to this point, we focused on the bacterial kingdom of the indigenous microbiota, but we cannot forget about the presence of others.

## 5 *T. gondii*-induced mycobial dysbiosis (Candida)

The microbiome is composed of many microbial taxa (bacteria, fungi, viruses, Archaea, protozoa), yet most studies still largely focus on measures of bacteria. Surprisingly, the fungi, which are taxonomic powerhouses and potent instigators of dysbiosis, are often overlooked (Severance, 2023). During times of good health, the body’s fungal residents, known as the mycobiome, exist in harmony with other microbial taxa. Many fungal species, however, are opportunistic, and as pathogens, will take over when the bacterial microbiome becomes dysbiotic (Kim and Sudbery, 2011; Forbes et al., 2018; CDC, 2019; MacAlpine et al., 2022; Underhill and Braun, 2022).

A role for opportunistic fungal species, such as *Candida albicans*, as drivers of *T. gondii-*controlled altered behavior and brain biochemistry has not been extensively studied. It seems logical that not only would an acute infection with *T. gondii* introduce a pro-inflammatory intestinal environment primed for bacterial dysbiosis and dominance by fungal pathogens, but that this dysbiotic state and related functional deficits would be maintained chronically until the dysbiosis was reversed (Saraav et al., 2021). In studies of fungal translocation in human neuropsychiatric disorders, levels of antibodies to the fungal species, *Saccharomyces cerevisiae* and *C. albicans*, were consistently elevated versus those from comparison groups (Severance et al., 2012; Severance et al., 2014; Severance et al., 2016; Hughes and Ashwood, 2018). In fact, *S. cerevisiae* antibodies have been routinely referred to as indices of GI inflammation and have been used to aid in the diagnosis of Crohn’s disease and to better understand the mycobiome of ulcerative colitis and other inflammatory diseases of the bowel (Torres et al., 2020; Gao and Zhang, 2021; Cimická et al., 2022).

The routes to the brain for fungal and parasitic taxa, independently or synergistically, have not been well defined. Numerous fungal species have been found in post-mortem brain tissue from individuals with amyotrophic lateral sclerosis, Alzheimer’s Disease, and Parkinson’s Disease (Alonso et al., 2015b; Alonso et al., 2015a; Pisa et al., 2015; Phuna and Madhavan, 2022). Microbial translocation generated locally in the GI tract has been hypothesized to lead to systemic, low-grade inflammation and a loss of integrity, not only of the blood-gut barrier, but also of the blood-brain barrier, thereby exposing the brain to access by microbes (Severance and Yolken, 2020a). Another means by which gut microbes, including fungi, might travel to the brain more directly is via microbial translocation and neuroinflammation along the vagus nerve (Thapa et al., 2023).

The concept of polymicrobial invasions of the brain, and a susceptibility of certain individuals to multiple neuropathogens, thus becomes a very relevant hypothesis (Carter, 2017). *T. gondii* and *C. albicans* are known to co-occur in individuals who are immune-suppressed, such as those with HIV (Bongomin et al., 2023). Immunosuppression or immune-modulation could also be gene-based, as a common hypothesis regarding the etiologies of psychiatric disorders, such as schizophrenia, is that these disorders are products of gene-by-environmental interactions (Severance and Yolken, 2020b). A genetic predisposition involving complement immune genes, combined with environmental exposures such as *T. gondii* and *C. albicans* infections, are hypothesized to elevate one’s risk of developing schizophrenia (Severance et al., 2021; Severance et al., 2023). It is also possible that previous infections may damage specific tissues and render them especially susceptible to future infections, as demonstrated in a mouse model of *T. gondii* chronic infection (Saraav et al., 2021). Furthermore, viral, fungal, and parasitic pathogens including *T. gondii*, can infect the same cell, together inactivate the immune system, and establish themselves as latent or chronic infections. Subsequent infections can lead to reactivation of the latent pathogens and T-cell exhaustion for the newly invading pathogens (Roe, 2022).

In conclusion, gut microbiome studies are currently flourishing, although we are still very much in the beginning stages of fully appreciating the complexities of this developing field. Studies to date have underestimated the potential role of fungi, which can quickly displace bacteria, create pro-inflammatory GI environments, and generate deleterious consequences on brain function and behavior. Acute and chronic infections of *T. gondii* very likely accelerate the deterioration of microbial balances, implicating a partnership with fungi that is mutually beneficial. Empirical studies to test for co-occurrences and functional co-associations of these exposures could help determine the extent that these infections are related.

## 6 Discussion

Toxoplasmosis poses a significant global health challenge affecting a substantial portion of the world’s population. The spectrum of its impact, from asymptomatic cases to severe neurological outcomes, underscores the complexity of this parasitic infection. In the intricacies of *T. gondii* infection and its consequential impact on behavior and neurological conditions, the gut microbiome emerges as a potentially vital piece, contributing to the complexity of this parasitic relationship (**Figure 1**).

The competency of *T. gondii* to infect any warm-blooded animals, including humans, and its ability to alter the host behavior for its own evolutionary advantages gained significant attention in the scientific community. Over the last 25 years, *T. gondii*-induced behavior changes captivated many scientists studying host-parasite relationships. Numerous studies investigated this sophisticated bond and revealed the involvement of different organ systems, including the nervous, immune, and endocrine systems. However, the exact mechanism is still waiting to be unveiled. Neuroinflammation, hormonal alterations, and modulation of neurotransmission stand as pieces of the puzzle, awaiting precise connections, connections that the gut microbiome and GBA may be able to clarify. Further research is needed utilizing germ free, antibiotic treated as well as probiotic treated host to assess the relationship of these individual domains to the microbiome during *T. gondii* infection. Mediation analysis has yet to be performed in order to disentangle the role of the microbiome in *T. gondii* induced behavior in the presence of the above discussed contributing domains. As mentioned, the genetic background and sex of the host as well as the genetic back ground of the parasite needs to be considered as potential cofounders along with infection route and the part of the intestine which was sampled.

As we delve into the gut-microbiome connection, the complexity of microbiota-gut-brain axis interactions adds a new layer to our understanding. Changes in microbiome composition during *T. gondii* infection, both acute and chronic, highlight the dynamic nature of these relationships. The gut-brain axis, facilitating communication between the central and enteric nervous systems, presents a previously underexplored dimension in the *T. gondii* saga.

The gut microbiome stands as a frontier for further exploration in the intricate web of *T. gondii* infection, behavioral alterations, and neurological consequences. Continued research in this field has the potential to unveil missing links, providing insights into potential therapeutic interventions and preventive strategies. The holistic comprehension of the interplay between parasites, host responses, and the microbiome opens new avenues for understanding the complex mechanisms governing the impact of *T. gondii* on both behavior and neurological health.
